# A bit toggling approach for AMBTC tamper detection scheme with high image fidelity

**DOI:** 10.1371/journal.pone.0230997

**Published:** 2020-04-16

**Authors:** Wien Hong, Dan Li, Der-Chyuan Lou, Xiaoyu Zhou, Chien-Hung Chang

**Affiliations:** 1 School of Electrical and Computer Engineering, Nanfang College of Sun Yat-Sen University, Guangzhou, China; 2 Department of Computer Science and Information Engineering, Chang Gung University, Taoyuan, Taiwan; 3 Stroke Center and Department of Neurology, Chang Gung Memorial Hospital, New Taipei, Taiwan; 4 College of Medicine, Chang Gung University, Taoyuan, Taiwan; Sam Houston State University, UNITED STATES

## Abstract

The existing tamper detection schemes for absolute moment block truncation coding (AMBTC) compressed images are able to detect the tampering. However, the marked image qualities of these schemes can be enhanced, and their authentication methods may fail to detect some special tampering. We propose a secure AMBTC tamper detection scheme that preserves high image fidelity with excellent detectability. In the proposed approach, a bit in bitmaps of AMBTC codes is sequentially toggled to generate a set of authentication codes. The one that causes the least distortion is embedded into the quantization levels with the guidance of a key-generated reference table (RT). Without the correct key, the same reference table cannot be constructed. Therefore, the proposed method is able to detect various kinds of malicious tampering, including those special tampering techniques designed for RT-based authentication schemes. The proposed method not only offers better image quality, but also provides an excellent and satisfactory detectability as compared with previous works.

## 1. Introduction

With the rapid development of image acquisition devices, digital images are more popular than before, and their applications are ubiquitous. Images are the most seen digital media over the Internet; however, modern computing technologies make digital images to be easily modified maliciously. Therefore, authenticating a received image has become crucial for numerous applications. Fragile watermarking [1–2] is a commonly used technique to protect the integrity of digital images by embedding fragile watermarks (or authentication codes, ACs) into images. Since the embedded ACs can be easily destroyed when modifying the marked image, the presence of tampering is detected and tampered trios can be located.

Fragile watermarking techniques can be applied to images of spatial [3–4] or compressed domains [5–18]. Because most of the images are stored and transmitted in compressed formats, the investigation of compressed-domain authentication techniques has received extensive research attention. A number of compressed domain authentication techniques such as vector quantization [5–6], joint photographic experts group (JPEG) [7–8], and absolute moment block truncation coding (AMBTC) [9–18] have been investigated. These techniques modify coefficients of compressed codes to embed authentication information, and the marked compressed codes can be decoded to generate an image that is similar to the original one. Since the AMBTC requires less computing cost but still offers acceptable compression ratio and image quality, it is applicable for applications demanding low-power consumption and limited transmission bandwidth such as devices for IoT. However, unprotected AMBTC codes are vulnerable to security threats such as malicious tampering. Therefore, protecting the transmitted AMBTC codes has become increasingly crucial for real applications. To protect the compressed codes from being tampered, several authentication techniques designed for AMBTC compressed images [9–18] have been investigated and gained wide attention due to the applicability of AMBTC.

The AMBTC compresses image blocks into trios. Each trio consists of two quantization levels and a bitmap. The quantization levels are used to record the intensities of image blocks while the bitmap is used to store the textures. The existing AMBTC authentication schemes embed ACs into either quantization levels or bitmaps. Hu et al. [11] propose a joint coding and authentication method for AMBTC codes. In their method, bitmaps are sub-divided, and ACs are embedded by matching the parity of sub-divided bitmaps. In Hu et al.’s another work [12], the length of ACs is designed to be adjustable for better embedding efficiency. Wu et al. [13] recognize that modifying the bitmap for AC embedment may cause larger distortions. Therefore, instead of bitmap, they embed ACs into quantization levels using a parity matching technique. Lin et al. [14] utilize the parity of bitmap to generate ACs and embed them into key-selected bits of quantization levels.

The early works [11–14] for authenticating AMBTC compressed codes mainly focus on the capability of detecting tampered trios. In addition to the detection performance, recent works [15–18] have paid more effort on improving the marked image quality by using reference table (RT) based embedding techniques [19–21] or adding the capability of recovery the tampered trios for some special applications [22]. Li et al. [15] present a RT-based AMBTC authentication scheme, in which two digits of base *λ* are embedded into a compressed trio using a RT of size *λ*×*λ*. Lin et al. [16] employ a hybrid scheme to embed ACs into smooth and complex blocks using different embedding strategies. Chen and Chang [17] notice that the bitmap in method [15] is unprotected, and present a new scheme such that the alteration of bitmap is detectable. Hong et al. [18] also propose a RT-based authentication scheme in which bitmaps are hashed to generate ACs. The ACs are then embedded into quantization levels using a RT-based technique [20].

While methods [15–18] remarkably protect the AMBTC compressed codes, some special designed tampering for the marked AMBTC trios may cause their authentication scheme failure. This is because the ACs generated from these methods are irrelevant to the codes they protect. As a result, the attackers may modify the marked trios in an intentional way to conceal the presence of tampering. Moreover, these methods all keep the bitmap intact when embedding the ACs. However, if quantization levels are employed to carry the ACs generated from the bitmap, toggling a bit in the bitmap might generate a different AC. The newly generated AC might lead to a smaller distortion than the one generated from the original bitmap. In light of this, this paper toggles bits in bitmaps to generate candidates of ACs. The best candidate that causes the least distortion is embedded into quantization levels using a key-protected RT-based embedding technique. Since the ACs are the hashed result of the toggled bitmap, any alteration of marked trios will extract incorrect ACs and thus the marked trios are fully protected.

The uniqueness of the proposed method is to present a method that perturbs bits of AMBTC’s bitmap to produce a set of selectable ACs. With the aids of the RT-based embedding technique, the AC with the smallest embedding distortion is securely embedded into the quantization levels. To the best of our knowledge, the proposed bitmap toggling technique is the first work to exploit the bitmap perturbation in combination with the RT-based embedding techniques in the discipline of AMBTC authentication. The experimental results demonstrate that the proposed method not only successfully detects any kinds of tampering, but also gives the best marked image quality when compared with prior works.

The rest of this paper is organized as follows. Section 2 briefly describes related works. Section 3 presents the proposed method, while Section 4 gives the experimental results. Concluding remarks are given in the last section.

## 2. Related works

In this section, the AMBTC compression technique will be briefly introduced. A RT-based method, which will be used as the embedding technique in the proposed method, is presented in this section. We also briefly introduce Chen and Chang’s work [17] in the last sub-section.

### 2.1 The absolute moment block truncation coding

The AMBTC [23], a variant of block truncation coding [24], is an efficient lossy compression technique proposed by Lema and Mitchell in 1984. To compress the original image *O*, the AMBTC partitions *O* into blocks ${\left\{O_{i}\right\}}_{i=1}^{N}$ of size *n*×*n*, where *N* is the total number of blocks. Each block *O*_*i*_ is represented by a lower quantization level *a*_*i*_, a higher quantization level *b*_*i*_, and a bitmap *B*_*i*_. Let *B*_*i*,*j*_ and *O*_*i*,*j*_ be the *j*-th bit of *B*_*i*_ and *j*-th pixel of *O*_*i*_, respectively. To obtain ${\left\{B_{i,j}\right\}}_{j=1}^{n\times n}$, the mean *m*_*i*_ of block *O*_*i*_ is calculated. If *O*_*i*,*j*_≤*m*_*i*_, *B*_*i*,*j*_ = 0. Otherwise, *B*_*i*,*j*_ = 1. The two quantization levels *a*_*i*_ and *b*_*i*_ are calculated by rounding the mean value of *O*_*i*,*j*_ with *B*_*i*,*j*_ = 0 and *B*_*i*,*j*_ = 1, respectively. Each block is compressed in the same manner, and the compressed trios ${\left\{a_{i},b_{i},B_{i}\right\}}_{i=1}^{N}$ are then obtained. To decompress the *i*-th trio {*a*_*i*_,*b*_*i*_,*B*_*i*_}, a block ${\left\{C_{i,j}^{}\right\}}_{j=1}^{n\times n}$ for storing decompressed results is initialized, where *C*_*i*,*j*_ is the *j*-th pixel of block *C*_*i*_. Then, pixels in ${\left\{C_{i,j}\right\}}_{j=1}^{n\times n}$ are sequentially visited. If *B*_*i*,*j*_ = 0, *C*_*i*,*j*_ = *a*_*i*_ is set. Otherwise, *C*_*i*,*j*_ = *b*_*i*_. Using the same procedure to decompress all trios, the decompressed AMBTC image ${\left\{C_{i}\right\}}_{i=1}^{N}$ is then obtained.

Here is an example to show the AMBTC compression method. Suppose *O*_*i*_ = [81,67,96,96; 70,19,96,54; 22,35,25,82; 92,59,97,33] is the original block to be compressed, where semicolons represent a row separator. The mean *m*_*i*_ of *O*_*i*_ is 64. Therefore, we have *B*_*i*_ = [1111; 1010; 0001; 1010], *a*_*i*_ = 35 and *b*_*i*_ = 86. Once the receiver has the compressed trio {*a*_*i*_,*b*_*i*_,*B*_*i*_} = {35,86,[1111; 1010; 0001; 1010]}, the decompressed block *C*_*i*_ = [86,86,86,86; 86,35,86,35; 35,35,35,86; 86,35,86,35] is obtained.

### 2.2 The reference table based embedding technique

**Fig 1:**
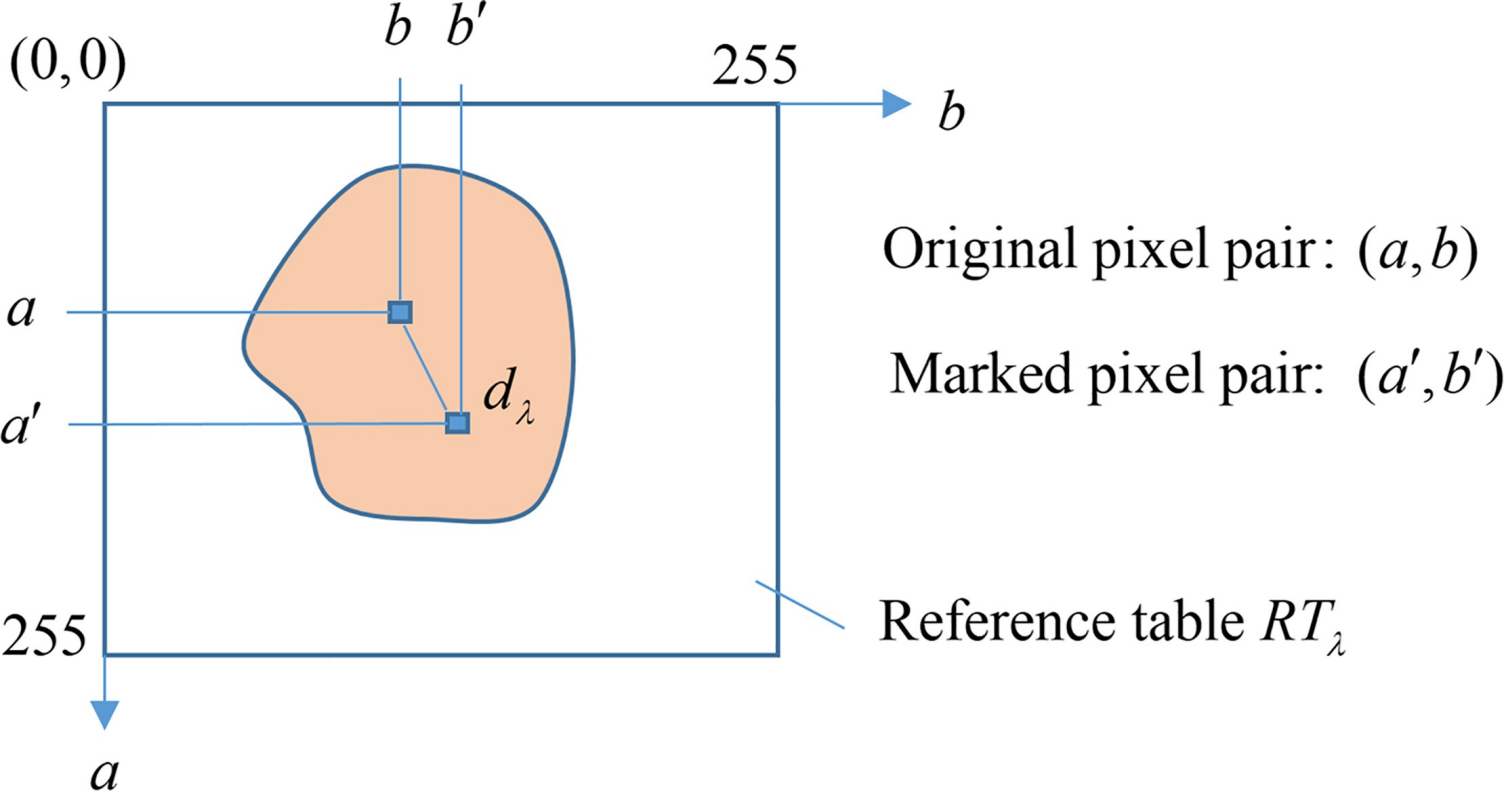
The illustration of RT-based embedding technique.

The RT-based embedding technique employs a pixel pair as an embedding unit, and embeds a secret digit *d*_*λ*_ of base *λ* by referencing a reference table *RT*_*λ*_. *RT*_*λ*_ is a matrix of size 256×256 filled with digits ranging from 0 to *λ*−1, and the element located in the *a*-th row and *b*-th column is denoted by *RT*_*λ*_(*a*,*b*). To embed *d*_*λ*_∈[0,*λ*−1] into a pixel pair (*a*,*b*), the neighboring elements of *RT*_*λ*_(*a*,*b*) are searched. The coordinate (*a*′,*b*′) satisfying *RT*_*λ*_(*a*′,*b*′) = *d*_*λ*_ and having the nearest distance to (*a*,*b*) is selected as the marked pixel pair. The embedded digit *d*_*λ*_ in coordinate (*a*′,*b*′) can be easily extracted by locating the element in (*a*′,*b*′) of *RT*_*λ*_, i.e., *d*_*λ*_ = *RT*_*λ*_(*a*′,*b*′). Fig 1 gives the illustration of RT-based embedding technique.

The design of reference table greatly affects the embedding performance. Several RT-based embedding methods [19–21] have been proposed during the past decade. Among these methods, the adaptive pixel pair matching (APPM) [20] achieves the lowest embedding distortion. The RT used in APPM is generated by using the following function

$$RT_{\lambda }(a,b)=\left(c_{\lambda }\times a+b\right)\mathrm{mod}\lambda ,$$ (1)

where *c*_*λ*_ is a *λ*-dependent coefficient, (*a*,*b*)∈Integers, 0≤*a*≤255 and 0≤*b*≤255. The often-used coefficients are *c*_4_ = 2, *c*_8_ = 3, *c*_16_ = 6, *c*_32_ = 7, *c*_64_ = 14 and *c*_256_ = 60. The full list of *c*_*λ*_ can be seen in [20].

**Fig 2:**
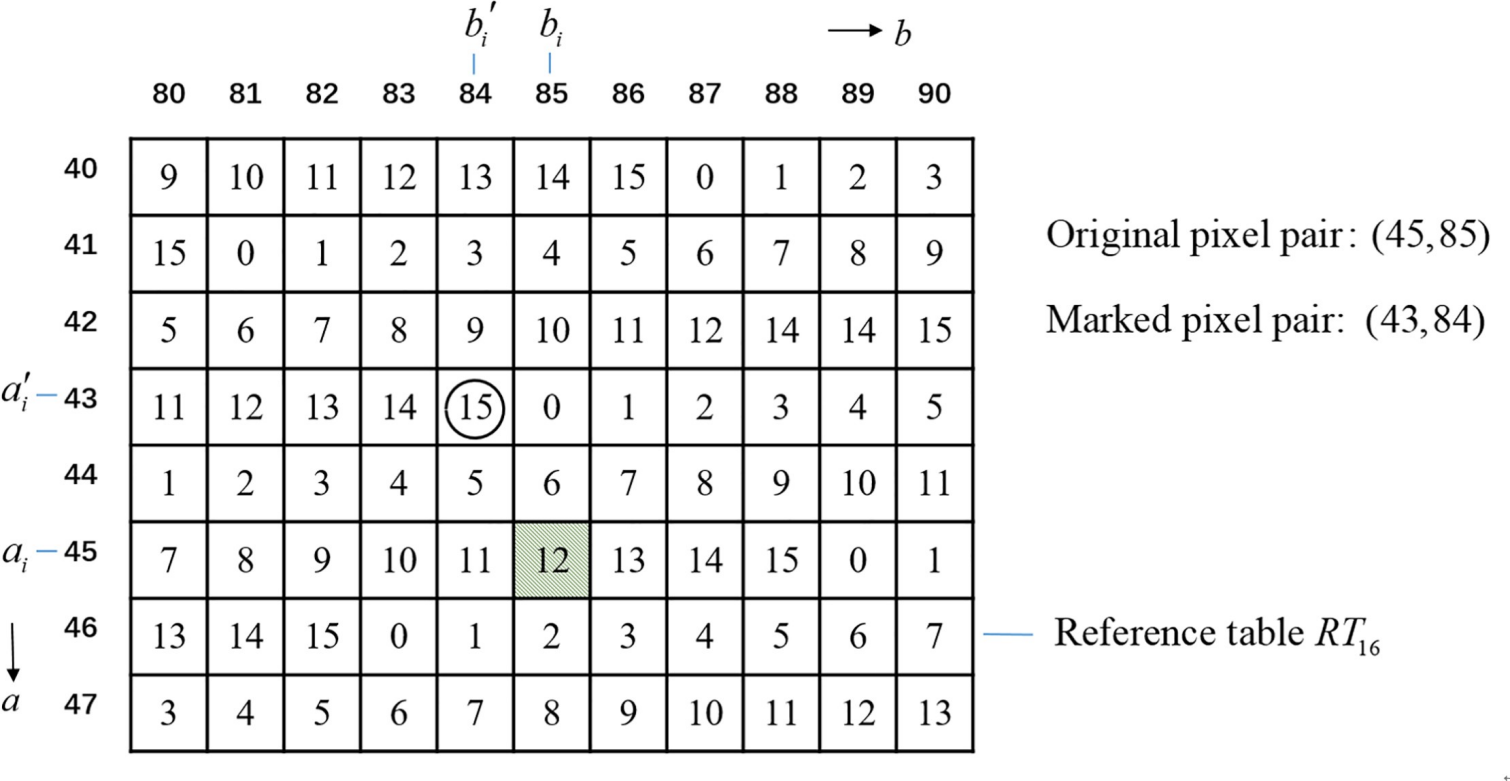
An example of the APPM method.

An example is given to show the APPM method as follows. Suppose the secret digit *d*_16_ = 15 of base 16 is to be embedded into the pixel pair (*a*,*b*) = (45,85). Fig 2 shows a portion of *RT*_16_, which can be generated from Eq (1). Since the element located in (43, 84) is 15, and the distance between (43, 84) and (45, 85) is the shortest, we obtain the marked pixel pair (*a*′,*b*′) = (43,84). Given the reference table *RT*_16_ and marked pixel pair (43, 84), the embedded digit *RT*_16_(43,84) = 15 can be extracted.

### 2.3 Chen and Chang’s scheme

In 2018, Chen and Chang [17] analyzed the weakness of Li et al.’s method [15] and proposed a secure authentication scheme for AMBTC compressed images. Li et al.’s scheme generates authentication digits using a random number generator, and embeds the generated digits into the quantization levels of the AMBTC codes by referencing a pre-designed reference matrix. Li et al.’s method offers a high visual quality of marked AMBTC image and is capable of detecting the alteration of quantization levels. However, the protection of bitmap is ignored in their method. In light of this, Chen and Chang proposes an improved version of Li et al.’s method by removing the aforementioned security concerns.

In Chen and Chang’s scheme [17], instead of using the random number generator, the authentication code *ac*_*i*_ is generated by calculating *ac*_*i*_ = (*rv*⊕*B*_*i*_)mod2^*ω*^, where *rv* is a random number, ⊕ denotes the exclusive-OR operator, and *ω* is the number of bits to be embedded into the *i*-th pair of the quantization level. Since the bitmap is used to generate the ACs, the alteration of bitmap would extract incorrect ACs. Therefore, the presence of tampering will be detected. The embedment and authentication procedures are similar to those of Li et al.’s method. See [17] for more details.

## 3. The proposed method

The existing RT-based AMBTC authentication schemes reduce image distortions to a large extent. However, the embedded ACs can be easily extracted from the marked trios once the used RT is known. As a result, one can tamper marked trios with some easy modifications to evade the detection by these methods. For example, [18] uses the APPM to embed ACs. Suppose (*a*′,*b*′) is a pair of marked quantization levels with the authentication code *d*_*λ*_ embedded. The embedded authentication code can be simply extracted by calculating *d*_*λ*_ = (*c*_*λ*_×*a*′+*b*′)mod*λ* (see Eq (1)). However, (*kλ*+(*c*_*λ*_×*a*′+*b*′))mod*λ* also gives the same *d*_*λ*_ for any integer *k*. As a result, one can tamper the marked image without being detected by [18] if adding $a_{i}^{\prime }$ and $b_{i}^{\prime }$ by *kλ*. The same problem also exists in methods [16] and [17]. Besides, the existing methods do not fully exploit the characteristic of bitmaps. In fact, since the ACs are generated from bitmaps, a slight modification of bitmaps could possibly introduces a smaller distortion after embedding the newly generated ACs. However, none of existing RT-based authentication schemes take the advantage of this property to reduce the distortion of marked images.

In this paper, we hash bitmaps and other information to generate ACs. A key-generated RT is used to further secure the authentication method. Since the same RT can only be reconstructed using the correct key, the tampering using aforementioned techniques can be easily detected. Moreover, a bit in the bitmap is toggled sequentially to generate a set of toggled bitmaps. The one that causes the least distortion after embedment will be selected as the marked bitmap. Therefore, the distortion is guaranteed to be smaller than or equal to the one without using this approach.

### 3.1 Generation of key-selected reference table

To prevent the leakage of embedded authentication codes, as has been discussed at the beginning of this section, we use a key *κ* to generate key-specified reference tables ${\left\{RT_{\lambda }^{\left(i\right)}\right\}}_{i=1}^{N}$. The element in the *a*_*i*_-th row and *b*_*i*_-th column of $RT_{\lambda }^{\left(i\right)}$ is generate by

$$RT_{\lambda }^{\left(i\right)}(a_{i},b_{i})=(RT_{\lambda }(a_{i},b_{i})+\sigma_{i})\mathrm{mod}\lambda ,$$ (2)

where *RT*_*λ*_(*a*_*i*_,*b*_*i*_) = (*c*_*λ*_×*a*_*i*_+*b*_*i*_)mod*λ*, and *σ*_*i*_ is a random integer generated by the key *κ*. Eq (2) successfully shifts the RT to the left circularly by *σ*_*i*_ mod*λ* units. The reference table $RT_{\lambda }^{\left(i\right)}$ will be used in the embedment of the *i*-th trio. The embedded ACs can only be successfully extracted with the correct key.

### 3.2 Generation and embedment of ACs

**Fig 3:**
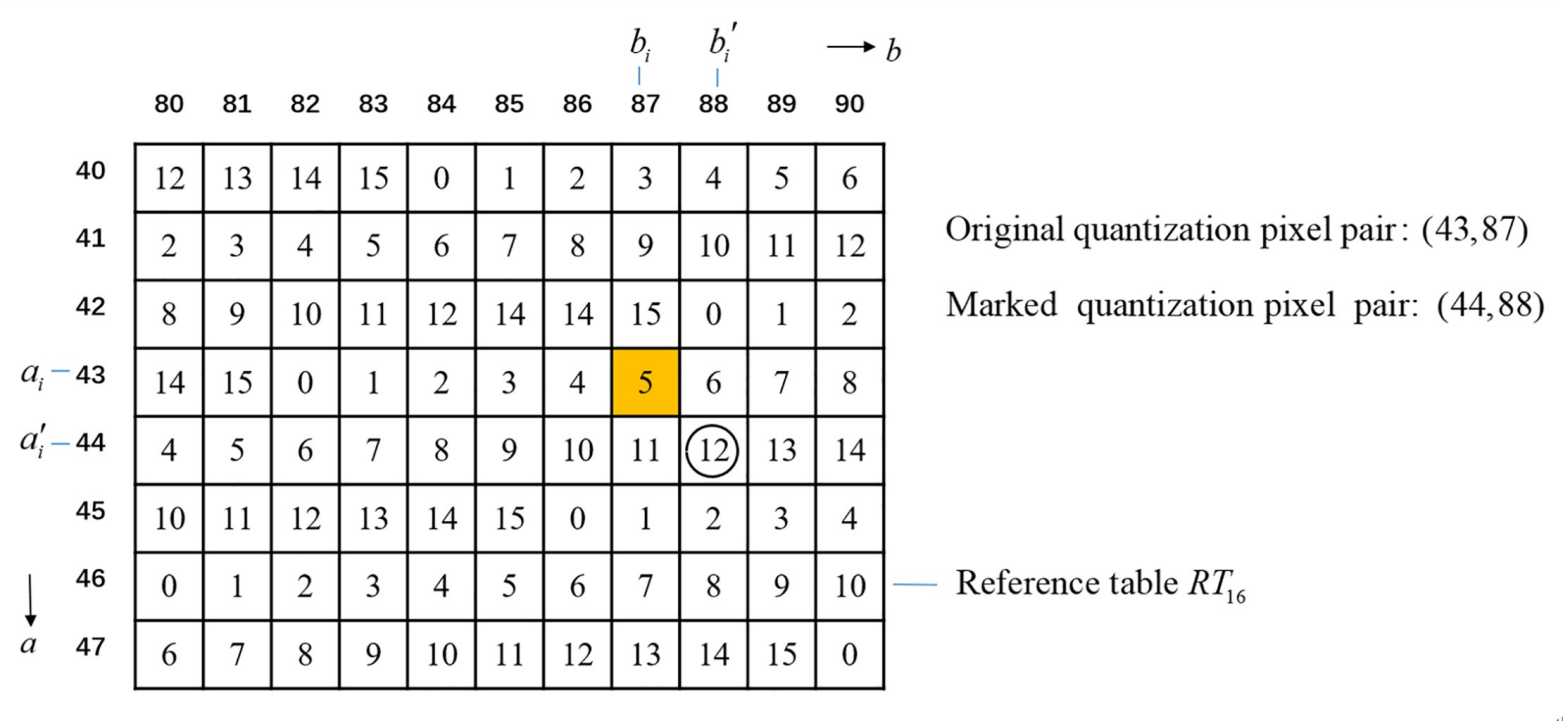
Example of the authentication code embedment.

Given the *i*-th AMBTC compressed trio (*a*_*i*_,*b*_*i*_,*B*_*i*_), we firstly generate a reference table $RT_{\lambda }^{\left(i\right)}$, as described in Section 3.1. Since $RT_{\lambda }^{\left(i\right)}$ is filled with digits ranging from 0 to *λ*−1, *w* =⌊log_2_*λ*⌋-bit information can be embedded into a trio. To generate a *w*-bit authentication code $ac_{i}^{w}$, we hash the bitmap *B*_*i*_, position information *i*, and the image identification *I*_*d*_ using the MD5 [25] hash function, and fold the hashed result into *w* bits using the exclusive-or operation. The authentication code $ac_{i}^{w}$ is then embedded into (*a*_*i*_,*b*_*i*_) to obtain the marked quantization level pair $\left(a_{i}^{\prime },b_{i}^{\prime }\right)$ using the APPM method under the guidance of $RT_{\lambda }^{\left(i\right)}$. The embedment of $ac_{i}^{w}$ can be formulated as

$$\mathrm{Minimize}:\;{\left(a_{i}-a_{i}^{\prime }\right)}^{2}+{\left(b_{i}-b_{i}^{\prime }\right)}^{2}$$ (3)

$$\mathrm{Subject}\;\mathrm{to}:\;ac_{i}^{w}={\mathrm{hash}}_{w}(B_{i},i,I_{d}),$$ (4)

$$RT_{\lambda }^{\left(i\right)}(a_{i}^{\prime },b_{i}^{\prime })=ac_{i}^{w},$$ (5)

where hash_*w*_(*x*) returns the decimal value of *w*-bit hashed result of *x*. Once Eqs (3)–(5) have been solved, the marked AMBTC trio $\left(a_{i}^{\prime },b_{i}^{\prime },B_{i}\right)$ can then be obtained. We continue the example given in Section 2.2 to illustrate the embedment of $ac_{i}^{w}$. Suppose the random generated integer is *σ*_*i*_ = 17763 and a portion of $RT_{16}^{\left(i\right)}$ is given in Fig 3. Let *a*_*i*_ = 43, *b*_*i*_ = 87 and $ac_{i}^{w}=12$. Because $RT_{16}^{\left(i\right)}(44,88)=12$ and (44,88) is the closest to (43,87), we have $\left(a_{i}^{\prime },b_{i}^{\prime })=(44,88\right)$.

### 3.3 Bit toggling technique

With the marked AMBTC trio $\left(a_{i}^{\prime },b_{i}^{\prime },B_{i}\right)$, the corresponding decompressed image block $\psi (a_{i}^{\prime },b_{i}^{\prime },B_{i})$ can be obtained. When compared with the original AMBTC block *ψ*(*a*_*i*_,*b*_*i*_,*B*_*i*_), the distortion occurs if $a_{i}\neq a_{i}^{\prime }$ or $b_{i}\neq b_{i}^{\prime }$. However, if the *k*-th bit of *B*_*i*_ is toggled, the AC generated from the toggled bitmap $B_{i}^{k}$ is likely to be very different from the one obtained from the un-toggled counterpart. After embedment, the decompressed block $\psi (a_{i}^{\prime },b_{i}^{\prime },B_{i}^{k})$ is possible to have a larger or smaller distortion than that of $\psi (a_{i}^{\prime },b_{i}^{\prime },B_{i})$ because the *k*-th bit of *B*_*i*_ is toggled. Therefore, bits in *B*_*i*_ can be toggled sequentially to find a toggled bitmap that causes the least distortion.

The bit toggling technique (BTT) is described as follows. Let $B_{i}^{k}$ be the bitmap *B*_*i*_ with *k*-th bit toggled and $B_{i}^{0}=B_{i}^{}$, 0≤*k*≤*n*×*n*. A total of *n*×*n*+1 authentication codes $ac_{i}^{w,k}={\mathrm{hash}}_{w}(B_{i}^{k},i,I_{d})$ can be generated. When embedding ${\left\{ac_{i}^{w,k}\right\}}_{k=0}^{n\times n}$ into (*a*_*i*_,*b*_*i*_) using APPM, a set of embedded regions ${\left\{a_{i}^{\prime k},b_{i}^{\prime k},B_{i}^{k}\right\}}_{k=0}^{n\times n}$ is obtained, and the mean square error between *ψ*(*a*_*i*_,*b*_*i*_,*B*_*i*_) and $\psi (a_{i}^{\prime k},b_{i}^{\prime k},B_{i}^{k})$ can be evaluated. The embedded trio that makes the decoded block having the least distortion is selected as the marked trio $\left(a_{i}^{\prime *},b_{i}^{\prime *},B_{i}^{*}\right)$. The solution to the aforementioned problem can be described using following optimization equations:

$$\mathrm{Minimize}:\;\mathrm{mse}\left(\psi (a_{i},b_{i},B_{i}),\psi (a_{i}^{\prime k},b_{i}^{\prime k},B_{i}^{k})\right)$$ (6)

$$\mathrm{Subject}\;\mathrm{to}:\;ac_{i}^{w,k}={\mathrm{hash}}_{w}(B_{i}^{k},i,I_{d}),$$ (7)

$$RT_{\lambda }^{\left(i\right)}(a_{i}^{\prime k},b_{i}^{\prime k})=ac_{i}^{w,k},$$ (8)

**Fig 4:**
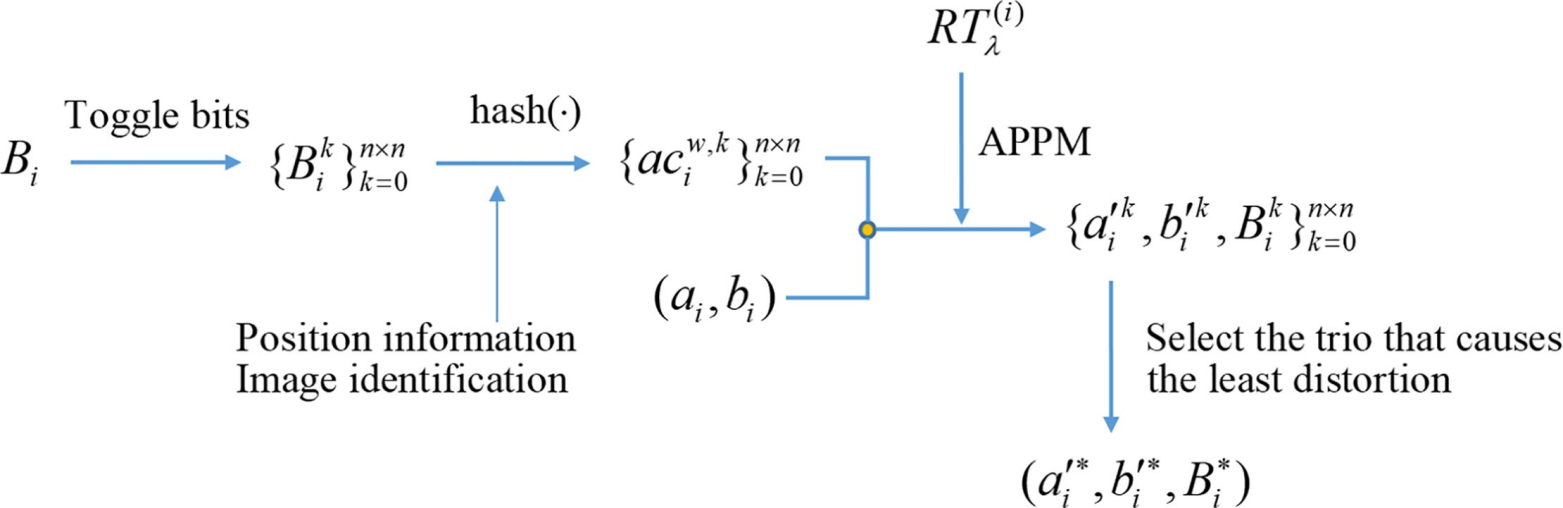
Schematic diagram of the bit toggling technique.

0≤k≤n×n, (9)

where $\mathrm{mse}\left(\psi (a_{i},b_{i},B_{i}),\psi (a_{i}^{\prime k},b_{i}^{\prime k},B_{i}^{k})\right)$ is a function for calculating the mean square error between *ψ*(*a*_*i*_,*b*_*i*_,*B*_*i*_) and $\psi (a_{i}^{\prime k},b_{i}^{\prime k},B_{i}^{k})$. All the embedment of trios is processed using the bit toggling technique, and the final marked trios ${\left\{a_{i}^{\prime *},b_{i}^{\prime *},B_{i}^{*}\right\}}_{i=1}^{N}$ are obtained. The schematic diagram of the BTT is shown in Fig 4.

We continue the example given in Section 3.2 to illustrate the proposed BTT. Suppose the generated ACs after bits toggled are ${\left\{ac_{i}^{4,k}\right\}}_{k=0}^{16}$ = {12, 7, 7, 15, 9, 12, 8, 2, 7, 9, 14, 13, 6, 8, 9, 0, 7}. Note that the zero-th AC (counting from zero) is generated from the un-toggled bitmap, i.e., $B_{i}^{0}=B_{i}^{}$. Among these ACs, the third one $ac_{i}^{4,3}=15$ and the 12-th one $ac_{i}^{4,12}=6$ both have the nearest distance to the element 5 located at (43, 87). Therefore, either one can be selected as the AC to be embedded into (*a*_*i*_,*b*_*i*_) = (43,87). Suppose we select the third one as the AC. As a result, the final marked trio should be $\left(a_{i}^{\prime *},b_{i}^{\prime *},B_{i}^{*})=(42,87,B_{i}^{3}\right)$.

### 3.4 The authentication procedures

**Fig 5:**
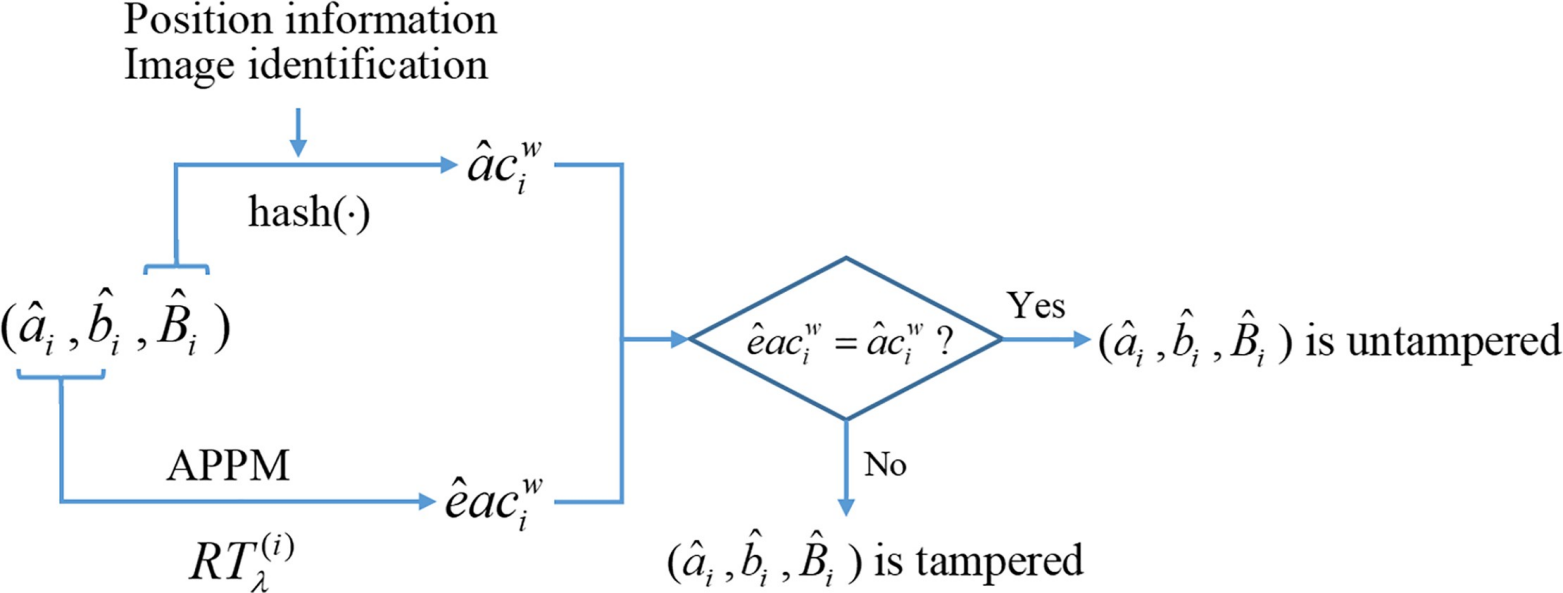
The schematic diagram of authentication procedures.

Let ${\left\{{\hat{a}}_{i},{\hat{b}}_{i},{\hat{B}}_{i}\right\}}_{i=1}^{N}$ be the AMBTC trios to be authenticated. Given the parameter *λ* and key *κ*, random integers ${\left\{\sigma_{i}\right\}}_{i=1}^{N}$ are generated and reference tables ${\left\{RT_{\lambda }^{\left(i\right)}\right\}}_{i=1}^{N}$ can be reconstructed. To authenticate the *i*-th trio $\left({\hat{a}}_{i},{\hat{b}}_{i},{\hat{B}}_{i}\right)$, the authentication code $\hat{a}c_{i}^{w}={\mathrm{hash}}_{w}({\hat{B}}_{i}^{},\hat{i},{\hat{I}}_{d})$ is generated. Meanwhile, the authentication code $\hat{e}ac_{i}^{w}$ embedded in quantization levels $\left({\hat{a}}_{i},{\hat{b}}_{i}\right)$ can be extracted by $\hat{e}ac_{i}^{w}=RT_{\lambda }^{\left(i\right)}({\hat{a}}_{i},{\hat{b}}_{i})$. If $\hat{a}c_{i}^{w}=\hat{e}ac_{i}^{w}$, the *i*-th trio is judged as an untampered trio. Otherwise, it is judged as a tampered one. The detection procedure is shown in Fig 5.

The aforementioned authentication procedures refer to as the coarse detection. Since the tampered regions are often contiguous, an untampered trio surrounded by tampered ones is likely to be also tampered. Therefore, a refined detection can be conducted by examining judged results of coarse detections. That is, if the upper and lower, left and right, upper-left and lower-right, or upper-right and lower-left trios of an untampered trio are judged as tampered ones, the untampered trio is re-judged as a tampered trio. This process is sequentially and repeatedly applied to all the trios, and the final detection result can be obtained.

## 4. Experimental results

**Fig 6:**
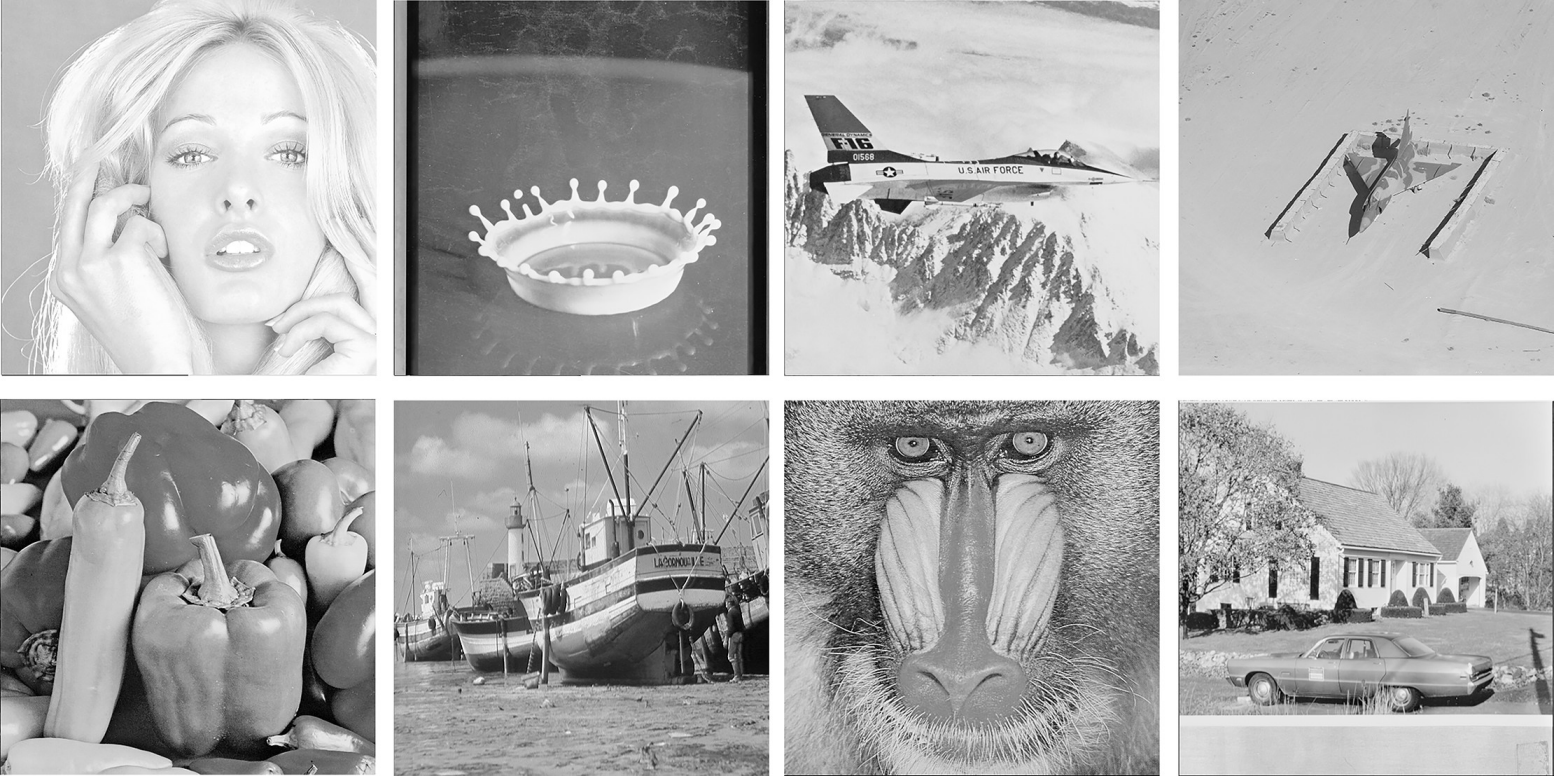
Eight test images of size 512×512. (a) Tiffany. (b) Splash. (c) Jet. (d) Airplane. (e) Peppers. (f) Boat. (g) Baboon. (h) House.

In this section, we conduct several experiments to demonstrate the effectiveness and applicability of the proposed method. We use eight grayscale images of size 512×512 shown in Fig 6 as the test images, and compress them to obtain the AMBTC trios. The AC embedment is then applied to obtain marked trios. The eight test images can be found in the USC-SIPI image database [26]. We also compare the performance of the proposed method with methods [15–18]. In all experiments, a block size of 4×4 is set.

We use the peak signal-to-noise ratio (PSNR) to measure the marked image quality. A higher PSNR indicates that the quality of marked image is more close to its unmarked version. In this paper, the PSNR measurement is defined by

$$\mathrm{PSNR}=10\log_{10}\frac{255^{2}}{\frac{1}{n\times n\times N}\sum_{i=1}^{n\times n\times N}{\left(x_{i}-x_{i}^{\prime }\right)}^{2}},$$ (10)

where *x*_*i*_ and $x_{i}^{\prime }$ represent the pixel values of images decompressed from the original and marked AMBTC trios, respectively.

### 4.1 Performance of the proposed method

**Fig 7:**
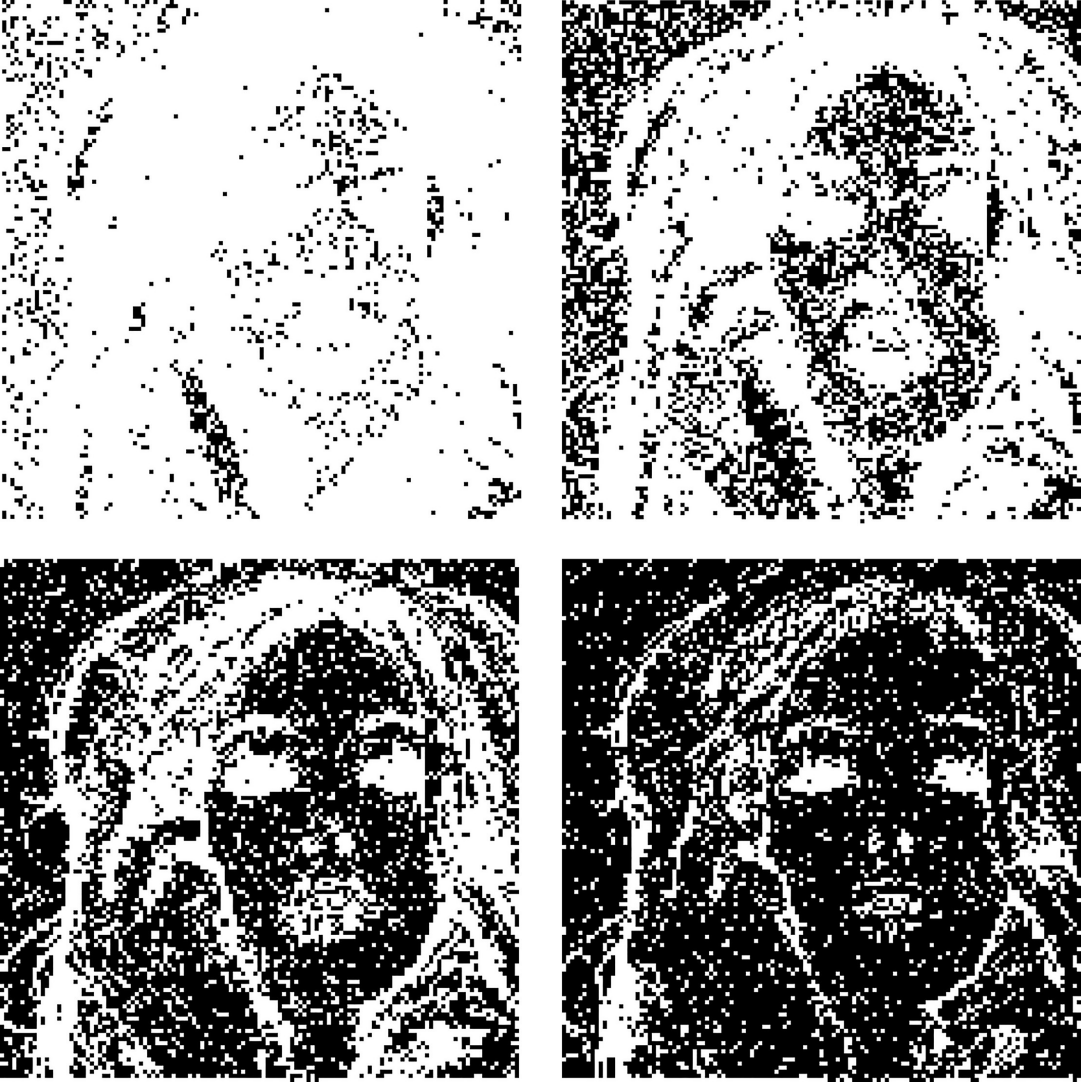
Effect of using the bit toggling technique. (a) *λ* = 4. (b) *λ* = 16. (c) *λ* = 64. (d) *λ* = 256.

It has been pointed out in Section 3 that for each trio, the distortion caused by using the BTT is always equal to or smaller than that of without using this technique. Fig 7(A)–7(D) demonstrate the effect of using BTT for *λ* = 4, 16, 64 and 256, respectively. In this figure, the black dots represent that the distortions of corresponding image blocks are reduced when the BTT is applied.

As seen from Fig 7, the number of black dots increases as *λ* increases. It means that the effect of using BTT becomes more apparent when more bits of ACs are embedded. The reason is that embedding a digit of larger base may cause more distortion, and toggling a bit in the bitmap has a greater chance to generate an AC that causes a less distortion. It is interesting to note that black dots distribute densely in smooth regions (e.g., Tiffany’s face and backgrounds) but distribute sparsely in complex regions (e.g., Tiffany’s hair). This is because toggling a bit in complex regions leads to more distortion than that of smooth ones. Therefore, the BTT is more effective when it is applied in smooth regions.

**Fig 8:**
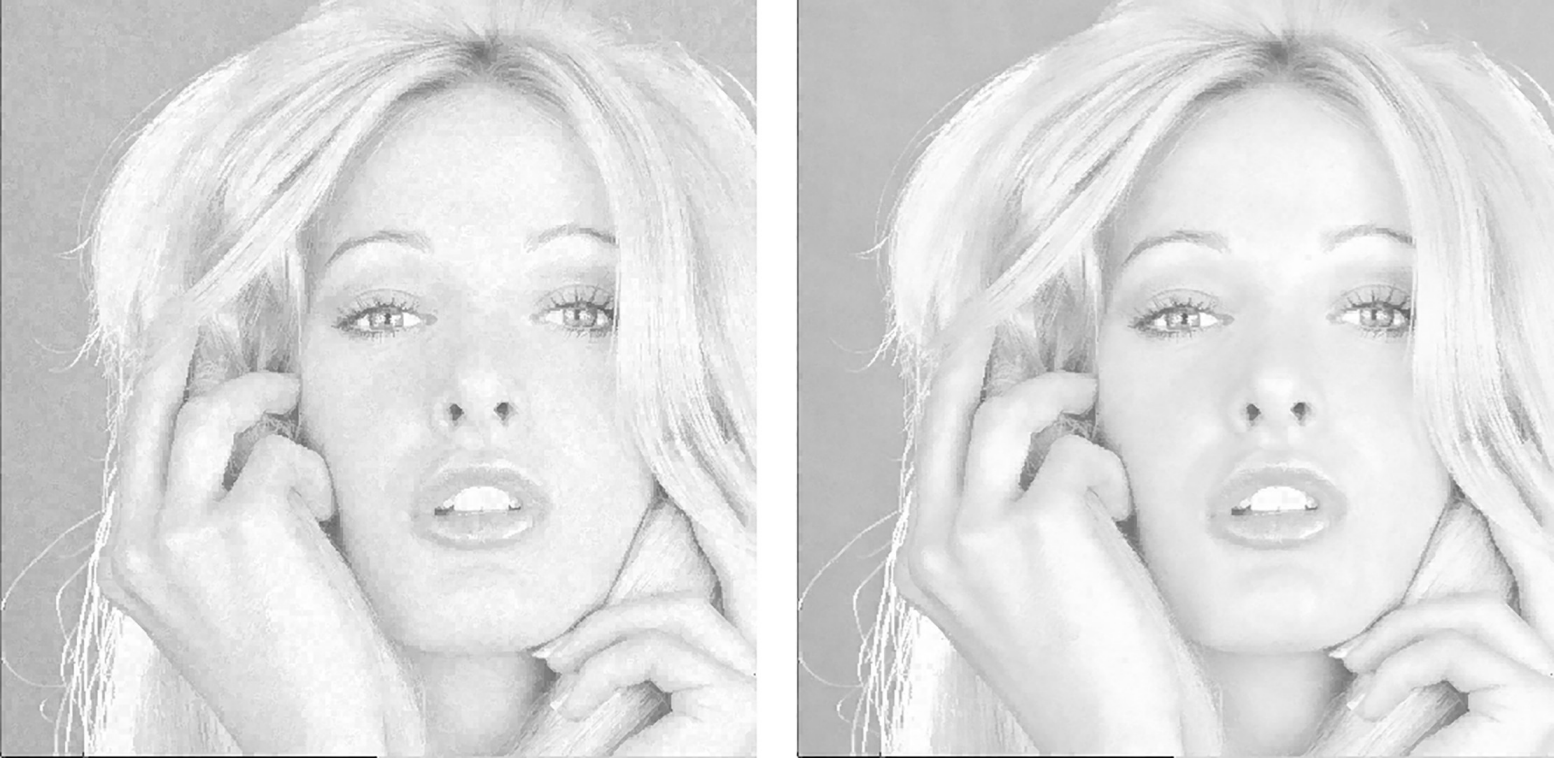
Image quality comparisons when using and without using the BTT. (a) Without BTT, PSNR = 34.89 dB. (b) With BTT, PSNR = 39.42 dB.

Fig 8 shows the visual quality comparisons of the enlarged marked Tiffany image when using and without using the BTT. In this experiment, we set *λ* = 256, which is equivalent to embedding 8-bit ACs into each trio. As seen from Fig 8(A) where the BTT is not applied, apparent noises distribute in smooth parts of the image such as Tiffany’s face and backgrounds. However, with the application of BTT, no apparent noises are found, demonstrating the effectiveness in enhancing the image quality.

The PSNR comparisons of using and without using the BTT with different embedding bases are list in Table 1. The results show that the gain of PSNR is more significant for a larger *λ*, conforming to previous discussions. It is worth to note that the gain of PSNR for *λ* = 64 and *λ* = 256 of the Airplane image is the highest because smooth regions of this image are more than others. On the other hand, the Baboon image has the smallest PSNR improvement because it possesses the richest textures.

**Table 1:** PSNR comparisons of using and without using the BTT (in dB).

Base	Method	Tiffany	Splash	Jet	Airplane	Peppers	Boat	Baboon	House
λ = 4	**BTT**	52.45	52.59	52.84	52.41	52.28	52.33	52.36	52.72
	**w/o BTT**	52.23	52.24	52.17	52.12	52.25	52.29	52.36	52.14
	**Gain**	0.22	0.35	0.67	0.29	0.03	0.04	0.00	0.58
λ = 16	**BTT**	47.92	48.19	48.35	49.07	47.32	47.17	46.88	47.69
	**w/o BTT**	46.86	46.87	46.87	46.87	46.86	46.89	46.86	46.79
	**Gain**	1.06	1.32	1.48	2.20	0.46	0.28	0.02	0.90
λ = 64	**BTT**	43.48	44.43	43.71	45.75	42.97	42.15	41.23	42.45
	**w/o BTT**	40.91	40.97	40.94	40.95,	40.96	40.94	40.93	40.84
	**Gain**	2.57	3.46	2.77	4.80	2.01	1.21	0.30	1.61
λ = 256	**BTT**	39.42	40.65	39.07	41.64	39.11	37.90	36.04	37.47
	**w/o BTT**	34.89	34.97	35.03	35.02	35.04	35.04	35.01	34.94
	Gain	4.53	5.68	4.04	6.62	4.07	2.86	1.03	2.53

**Fig 9:**
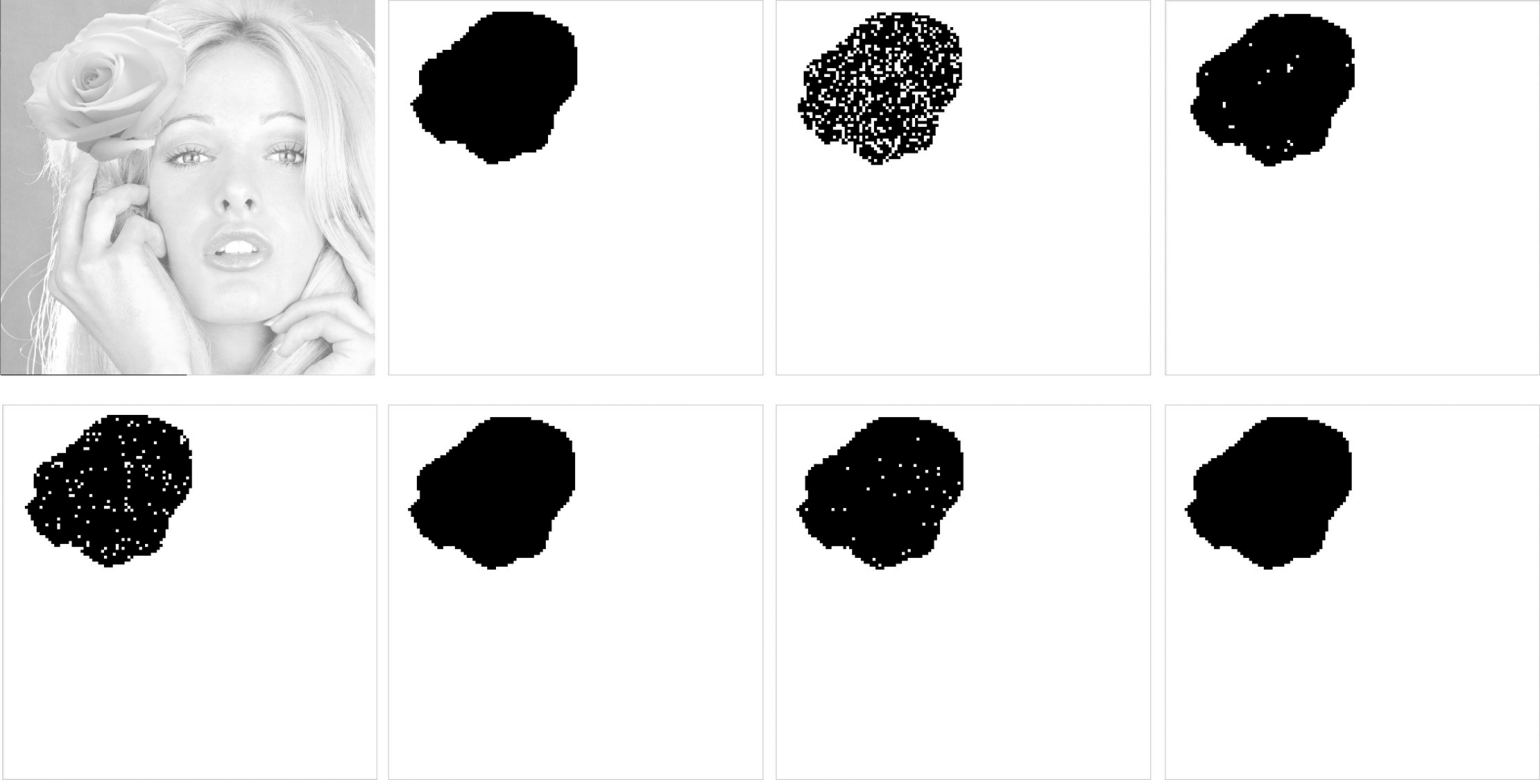
Detectability comparisons of coarse and refined detections for various *λ*. (a) Tampered image. (b) Tampered area. (c) Coarse detection *λ* = 4. (d) Refined detection *λ* = 4. (e) Coarse detection *λ* = 16. (f) Refined detection *λ* = 16. (g) Coarse detection *λ* = 64. (h) Refined detection *λ* = 64.

Fig 9 shows the performance of coarse and refined detections of the proposed method when setting *λ* = 4, 16 and 64. We tamper the Tiffany image by splicing a rose on Tiffany’s hat (Fig 9(A)), and the tampered region is shown in Fig 9(B). Fig 9(C)–9(H) give the detection results for different *λ*, where the black dots represent that the corresponding blocks are reported as tampered ones.

The experiments show that for all coarse detections, some white spots appear in the tampered regions due to hash collisions. In fact, the hash collision rate of the coarse detection is 1/2^*λ*^. Therefore, the number of white spots decreases as *λ* increases. Nevertheless, with the application of refined detection, white spots are successfully eliminated. As seen in Fig 9(F) and 9(H), the refined detections perform equally well when setting *λ* = 16 and *λ* = 64 because no white spots exist for both settings. However, setting *λ* = 64 causes larger distortions than those of *λ* = 16. Therefore, we recommend setting *λ* = 16 to achieve a balance between image quality and detectability.

### 4.2 Image quality comparisons with other works

In this section, we compare the proposed method with other related works, including Hong et al.’s [18], Chen et al.’s [17], Lin et al.’s [16], and Li et al.’s [15] methods. To make a fair comparisons, the Hong et al.’s method [18] is implemented such that smooth and complex blocks carry an equal number of authentication bits. In [17] and [15], a reference table of size 4×4 is used so that each trio is capable of carrying ACs of 2 or 4 bits. In [16], we set the threshold equal to 7 to achieve the best image quality, as suggested in the original paper. Since the trios in methods [15–17] cannot carry ACs larger than 4 bits, we only compare the marked image quality when 2-bit and 4-bit ACs are embedded. The results are shown in Table 2.

**Table 2:** PSNR comparisons for various lengths of AC.

AC	Method	Tiffany	Splash	Jet	Airplane	Peppers	Boat	Baboon	House
**2 bits**	**Proposed**	52.45	52.59	52.84	52.41	52.28	52.33	52.36	52.72
	**[18]**	52.18	52.26	52.12	52.09	52.25	52.28	52.39	52.18
	**[17]**	51.09	51.02	51.14	51.17	51.10	51.10	51.07	51.14
	**[16]**	48.33	47.74	49.29	48.35	47.41	48.30	50.17	49.84
	**[15]**	48.12	51.07	51.16	51.10	51.13	51.13	51.14	51.17
**4 bits**	**Proposed**	46.92	46.86	46.79	49.07	46.79	46.79	46.88	46.87
	**[18]**	46.78	46.89	46.90	46.89	46.89	46.88	46.84	46.74
	**[17]**	44.35	44.36	44.28	44.24	44.39	44.36	44.38	44.33
	**[16]**	44.58	44.29	45.41	44.63	43.71	44.22	45.28	45.62
	**[15]**	44.43	44.48	44.38	44.59	44.36	44.23	44.28	44.30
**6 bits**	**Proposed**	43.48	44.43	43.71	42.97	42.97	42.15	41.23	42.45
	**[18]**	40.92	40.93	40.95	40.95	40.93	40.96	40.96	40.93
**8 bits**	**Proposed**	39.42	40.65	39.07	41.64	39.11	37.90	36.04	37.47
	**[18]**	34.93	35.04	34.92	35.05	35.00	35.02	35.00	34.97

As shown in the table, the image qualities of all test images of [16] are the lowest when 2-bit ACs are embedded. The reason is that the RT they used is a Sudoku table, which introduces more distortions than the one constructed using Eq (1). Methods [15] and [17] offer almost identical image qualities because both methods adopt the same embedding technique. However, Chen and Chang’s method [17] is more secure than that of Li et al.’s method [15] because the bitmaps in [17] are fully protected. The Hong et al.’s method [18] provides better PSNR than that of [15–17]; however, their method cannot detect some types of special tampering, as has been described in Section 3. Obviously, the proposed method offers the best image quality for all test images due to the proposed BTT. Moreover, the length of AC of the proposed method is adjustable and can be extent to embed 3- or 4-bit ACs per trio, while methods [15–17] cannot. Notice that the proposed method provides significantly better image quality than that of [18], especially for larger length of AC. For example, when the lengths of AC are 6 (*λ* = 64) and 8 (*λ* = 256), the improvements in PSNR of the Tiffany image are 43.48−40.92 = 2.56 dB and 39.42−34.93 = 4.49 dB, respectively. Besides, the proposed method is more secure in protecting the AMBTC trios than that of [18], as will be presented in the next subsection.

### 4.3 Detectability comparisons with other works

**Fig 10:**
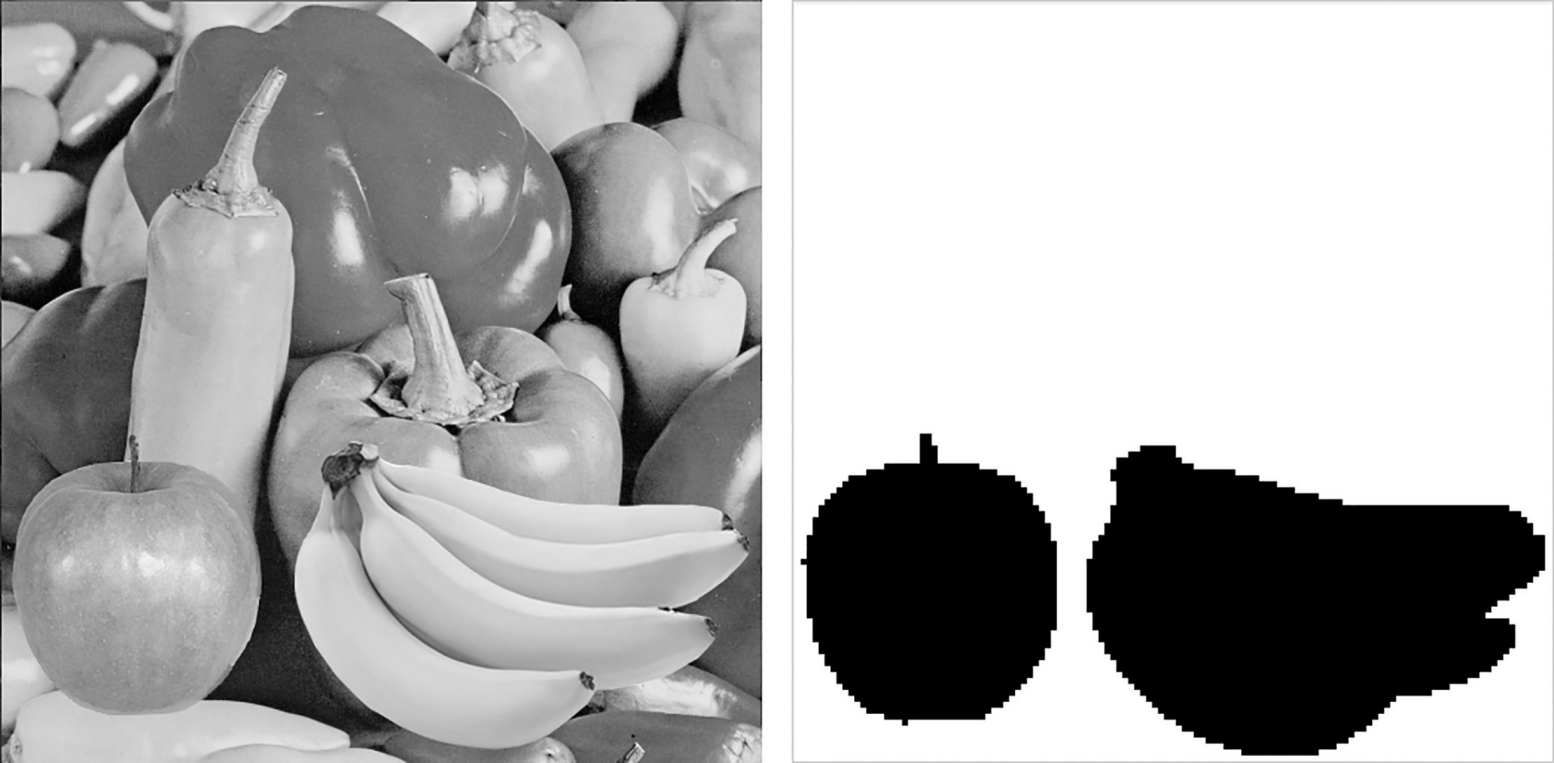
Tampered peppers image and tampered regions. (a) Tampered image. (b) Tampered Regions.

This section demonstrates the detectability comparisons of Hong et al.’s [18], Chen et al.’s [17], Lin et al.’s [16], and the proposed methods. In this experiment, the marked trios of Peppers image are tampered so that the tampered decompressed image exhibits an apple and bananas on the Peppers image, as seen in Fig 10(A). Fig 10(B) shows the tampered regions, where tampered blocks are represented by black color. To make a fair comparison, all methods are implemented to embed 4-bit AC into each trio (*λ* = 16). When implementing the Lin et al.’s method [16], the threshold used to distinguish smooth and complex blocks is set to 7, as suggested in their original paper.

To splice the Apple image onto the marked Peppers image, the original Apple image *I*^*A*^ is partitioned into blocks ${\left\{I_{i}^{A}\right\}}_{i=1}^{\tau }$, and temporarily placed onto the Peppers image, where *τ* is the total number of blocks of *I*^*A*^. Each block $I_{i}^{A}$ is then compared with all the blocks of the marked Peppers image ${\left\{I_{i}^{P}\right\}}_{i=1}^{N}$. The block $I_{i}^{P*}$ in ${\left\{I_{i}^{P}\right\}}_{i=1}^{N}$ that is the most similar to $I_{i}^{A}$ is then used to replace $I_{i}^{A}$. We denote this type of tampering as Type 1 tampering. To tamper the marked Peppers image by the Bananas image *I*^*B*^, the blocks ${\left\{I_{i}^{B}\right\}}_{i=1}^{\varphi }$ of *I*^*B*^ are temporarily replaced blocks ${\left\{I_{i}^{P}\right\}}_{i=1}^{\varphi }$ of the Peppers image, where *φ* is the total number of blocks of *I*^*B*^. Let ${\left\{a_{i}^{P},b_{i}^{P},B_{i}^{P}\right\}}_{i=1}^{\varphi }$ and ${\left\{a_{i}^{B},b_{i}^{B},B_{i}^{B}\right\}}_{i=1}^{\varphi }$ be the trios of ${\left\{I_{i}^{P}\right\}}_{i=1}^{\varphi }$ and ${\left\{I_{i}^{B}\right\}}_{i=1}^{\varphi }$, respectively. The quantization levels $\left(a_{i}^{B},b_{i}^{B}\right)$ are then adjusted to find integers *k*_1,*i*_ and *k*_2,*i*_ such that ${\left(16k_{1,i}+a_{i}^{P}-a_{i}^{B}\right)}^{2}+{\left(16k_{2,i}+b_{i}^{P}-b_{i}^{B}\right)}^{2}$ is the smallest. In this way, the final spliced Bananas image is very similar to its original image but identical ACs can be extracted from $\left(a_{i}^{P},b_{i}^{P}\right)$ and $\left(16k_{1,i}+a_{i}^{P},16k_{2,i}+b_{i}^{P}\right)$ using the RT-based embedding method. We denote this type of tampering as Type 2 tampering.

**Fig 11:**
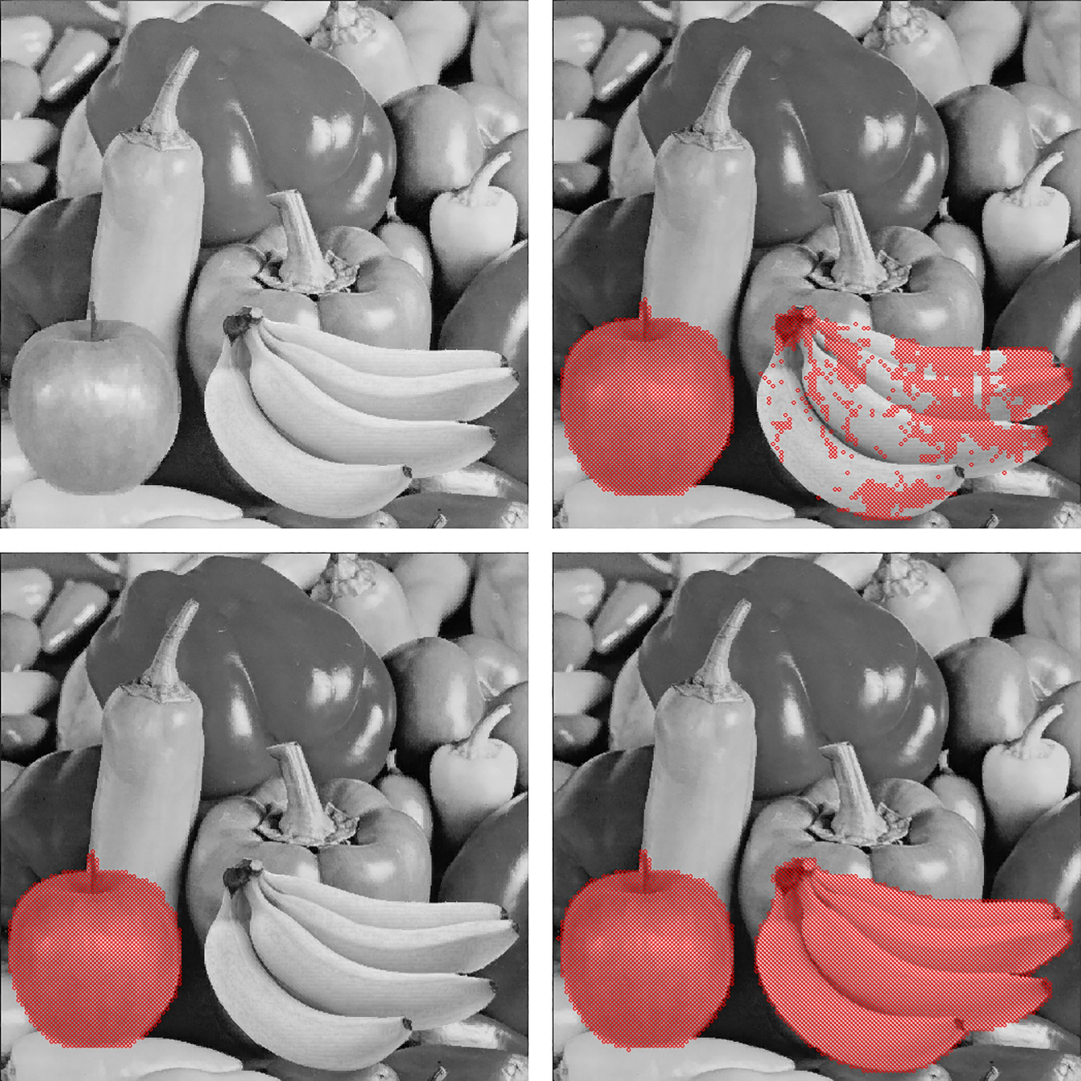
Detectability comparisons of the proposed and other methods. (a) Detection results of [18]. (b) Detection results of [16]. (c) Detection results of [17]. (d) Detection results of the proposed method.

The detection results are shown in Fig 11(A)–11(D), where the red circles represent that the corresponding blocks are reported as tampered ones. As shown in Fig 11(A), both the spliced Apple and Bananas images cannot be detected using the Hong et al.’s method [18]. The reason is that their method does not utilize the location information of trios to generate ACs. Therefore, the Apple image composed by the most similar blocks of marked Peppers image cannot be detected.

The spliced Bananas image also cannot be detected by the Hong et al.’s method [18] because the trios ${\left\{16k_{1,i}+a_{i}^{P},16k_{2,i}+b_{i}^{P},B_{i}^{B}\right\}}_{i=1}^{\varphi }$ of the spliced Bananas image always satisfy the equality $(c_{16}\times a_{i}^{P}+b_{i}^{P})\mathrm{mod}16=(c_{16}\times (16k_{1,i}+a_{i}^{P})+16k_{2,i}+b_{i}^{P})\mathrm{mod}16$. Therefore, the same ACs can be extracted from the marked and tampered images, leading to a failure in detecting the manipulated image. Similar problems also happen in [17], where the spliced Bananas image cannot be detected. Notice that the spliced Bananas image can be partially detected by the Lin et al.’s method [16]. In fact, the ACs of [16] of the detected tampered blocks are embedded in bitmaps because these blocks are classified as complex ones. For those smooth blocks where the ACs are embedded in quantization levels, the presence of tampering cannot be detected because adding or subtracting the marked quantization levels by multiples of 16 will extract the same authentication codes. Nevertheless, the proposed method successfully detects both spliced Apple and Bananas images (Fig 11(D)), indicating that the proposed method is capable of detecting both types of malicious tampering.

**Fig 12:**
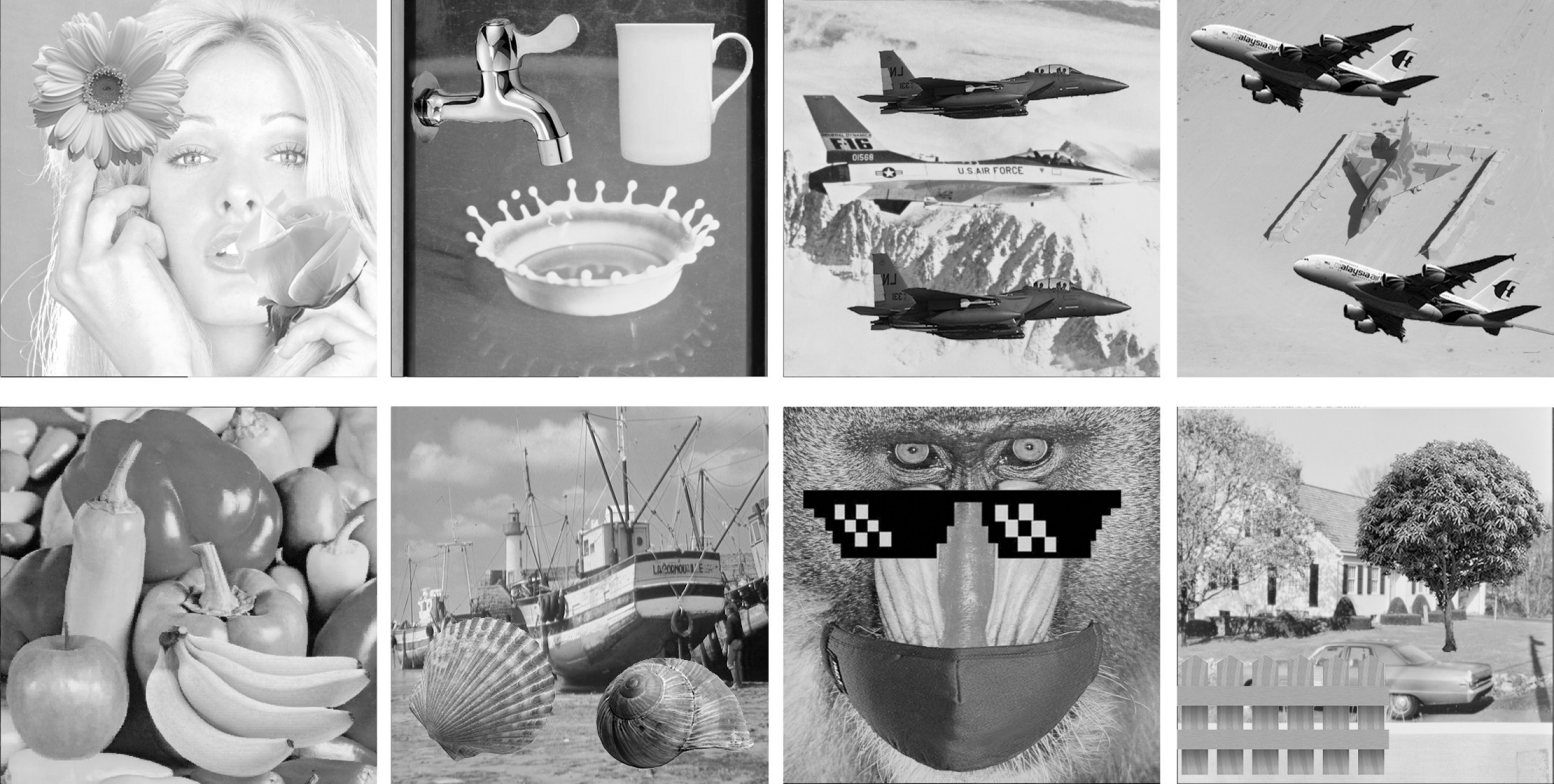
Eight tampered test images of size 512×512. (a) Tiffany. (b) Splash. (c) Jet. (d) Airplane. (e) Peppers. (f) Boat. (g) Baboon. (h) House.

To further validate the detection capability of the proposed method, we apply Type 1 and Type 2 tampering approaches to tamper the eight test images and compare the results with those of [18], [16] and [17]. The tampered images are shown in Fig 12. In these experiments, we splice a daisy, a faucet, an upper jet, an upper airplane, an apple, a seashell, a glasses, and a tree onto Fig 12(A)–12(H), respectively using the Type 1 tampering approach. Moreover, we also splice a rose, a cup, a lower jet, a lower airplane, bananas, a snail, a mask, and a fence onto Fig 12(A)–12(H), respectively using Type 2 tampering approach.

**Fig 13:**
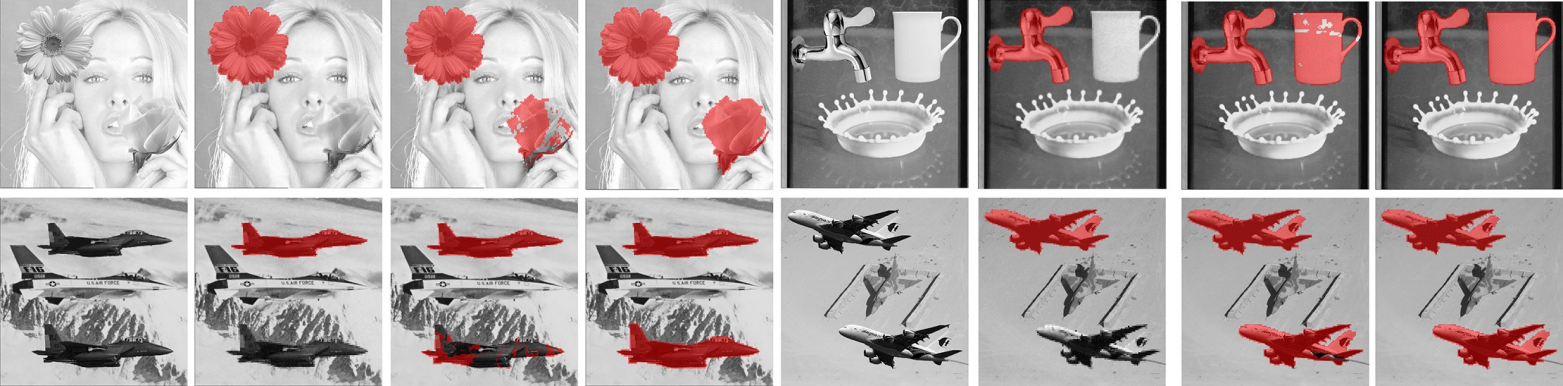
Detection results of [18], [16], [17] and the proposed method for smooth images. (a)-(d) Tiffany image. (e)-(h) Splash image. (i)-(l) Jet image. (m)-(p) Air-plane image.

**Fig 14:**
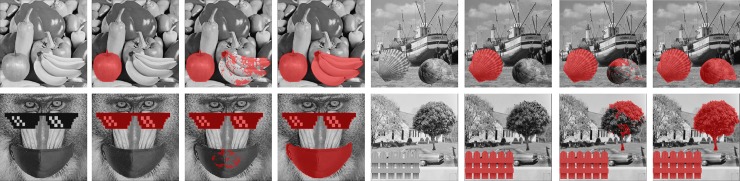
Detection results of [18], [16], [17] and the proposed method for complex images. (a)-(d) Peppers image. (e)-(h) Boat image. (i)-(l) Baboon image. (m)-(p) House image.

We apply the proposed method and other related works [16–18] to eight test images to show the influence of image smoothness on the detectability. In these test images, Tiffany, Splash, Jet and Airplane are smoother than Peppers, Boat, Baboon and House. Fig 13 shows the detection results of smooth images whereas Fig 14 gives the results of complex ones. The red dots represent that the corresponding blocks are reported as tampered.

When authenticating the smooth images, the Hong et al.’s method [18] neither detects Type 1 nor Type 2 tampering. Lin et al.’s method [16] and Chen et al.’s method [17], are able to detect Type 1 tampering; however, they fail or partially fail to detect the tampering of Type 2. In contrast, both types can be detected by the proposed method (See Fig 13(A)–13(P)).

The detection results of complex images show the similar trends, as can be seen in Fig 14(A)–14(P). In fact, because all the compared methods embed equally amount of data into both smooth and complex blocks, the tamper detection rate is only relevant to the number of embedded bits, regardless of the image complexity. Therefore, the experiments on the smooth and complex images reveal the similar results. Notice that Lin et al.’s method [16] still cannot fully detect the Type 2 tampering. Since their method uses a threshold to control where should the authentication code be embedded, the setting of the threshold may affect the detection result. In this experiment, the threshold is also set to 7. If the threshold is set to 255, the tampered regions should be totally undetectable. However, this setting will significantly degrade the marked image quality. Nevertheless, the proposed method effectively detects both tampering types of the complex images.

In light of the experimental results shown above, the proposed method offers a better detectability and marked image quality, meaning that proposed method is more suitable for authenticating the AMBTC compressed images.

## 5. Conclusions

In this paper, a new AMBTC authentication method with high image quality and efficient detectability is proposed. To improve the marked image quality, a bit in the bitmap that causes the least distortion after embedment is selected and toggled to generate the ACs. The introduced embedding distortion of the bit toggling technique is guaranteed to be smaller than or equal to that of Hong et al.’s method. To enhance the security, a key-specified reference table is used for guiding the embedment of ACs. Prior to the extraction of ACs, the same reference table that used in the embedding phase has to be constructed. Therefore, without the correct key, the embedded ACs cannot be extracted and attackers would be impossible to design undetectable tampered trios. The experiments show that the marked image quality of the proposed method is the highest when compared with prior related works. Moreover, the proposed method is able to detect all kinds of tampering while other methods can only detect some of them.
